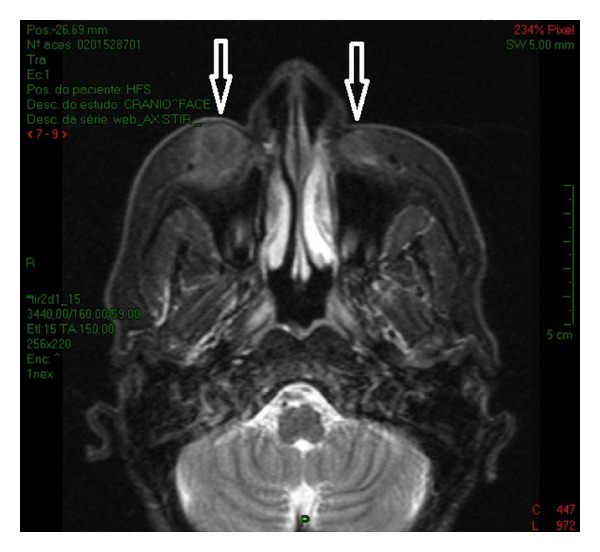# The Therapeutic Benefit of Allopurinol in the Treatment of Foreign Body Granulomas Caused by Polymethylmethacrylate Microspheres

**DOI:** 10.1155/2012/945205

**Published:** 2012-12-27

**Authors:** Luisa Kelmer Côrtes de Barros Silveira, Felipe Ladeira de Oliveira, Thais de Barros Castro Alves, Maria Lourdes Candela Rambaldi, Felipe Cupertino de Andrade, Santuzza de Carvalho Kelmer, Fabio Cuiabano Barbosa

**Affiliations:** General Dermatology Department, Dermatology Clinic Dr. Fabio Cuiabano Barbosa, Rua Visconde de Pirajá 330, Ipanema, 22410-000 Rio de Janeiro, RJ, Brazil

## Abstract

Injectable polymethylmethacrylate (PMMA) microspheres are nonbiodegradable and too large for macrophage phagocytosis. There are several complications possible to happen, like chronic nonspecific inflammatory reactions, lip stiffness, infection, and granulomas. The occurrence of granulomas can lead to a not aesthetic result, making some extreme changes in the patient's life. The objective of this case report is to describe the successful treatment of foreign body granulomas caused by polymethylmethacrylate microspheres using allopurinol, an innovative therapy for this condition.

## 1. Introduction

Injectable polymethylmethacrylate (PMMA) is composed of 80% bovine collagen and a suspension of 20% nonreabsorbable PMMA. In Brazil, one of the types used is Metacrill (Nutricel Laboratórios, Rio de Janeiro, Brazil), made of polymethylmethacrylate microspheres (30%) ranging from 40 to 60 mm in diameter, which is suspended in a nonabsorbable chemical colloid composed of carboxymethylcellulose [1].

The action mechanism consists on the collagen carrier being degraded by the body within 1–3 months, so the collagen recently formed is deposited. It is encapsulated and engulfed by the remaining PMMA particles, which are extremely long lasting and may be permanent [2]. The PMMA microspheres are nonbiodegradable and too large for macrophage phagocytosis. 

There are several complications possible to happen, like chronic nonspecific inflammatory reactions, lip stiffness, infection, nodules, tissue necrosis, lymphedema, and granulomas. The formation of a massive giant cell around an implant indicates foreign body granuloma. Granuloma is a multinucleated giant cell surrounded by palisading macrophages enveloped in a halo of lymphocytes [3]. The occurrence of granulomas can lead to a not aesthetic result, making some extreme changes in the patient's life.

## 2. Case Representation

A patient, 56-year-old, female, born in and resident of the city of Rio de Janeiro, went to the ambulatory of a dermatology clinic complaining of facial swelling for one month.

She states that in August 2000 she consulted a plastic surgeon for cosmetic purposes and underwent a filling procedure of the nasolabial folds, glabellar region, and lips with PMMA.

**Figure 1:**
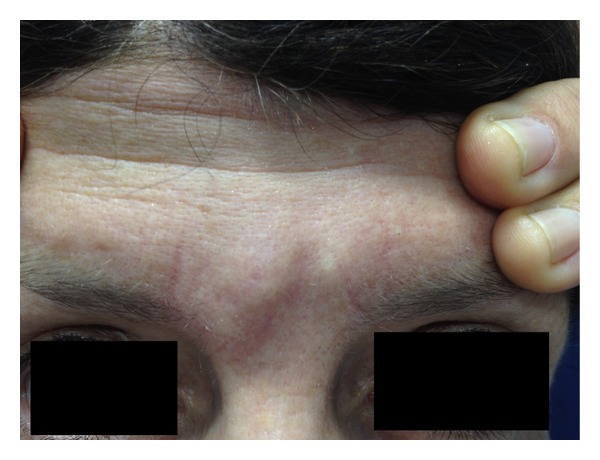


**Figure 2:**
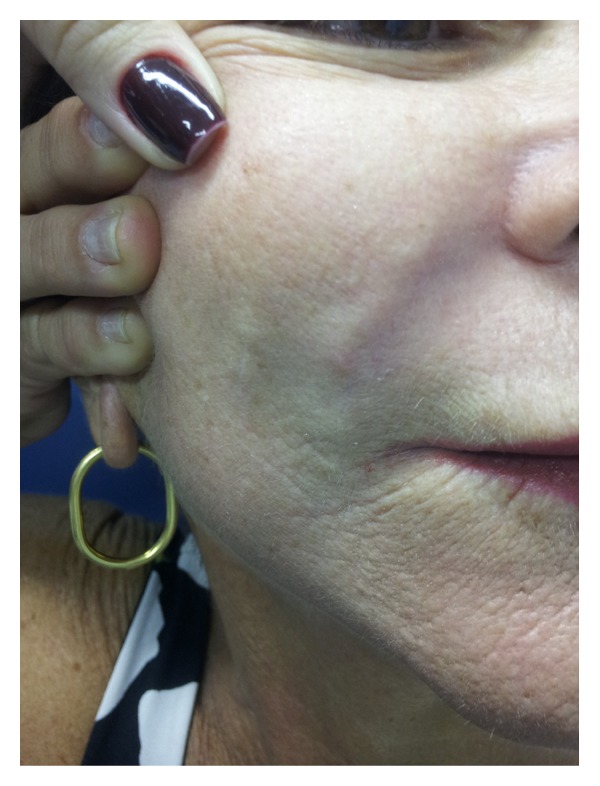


**Figure 3:**
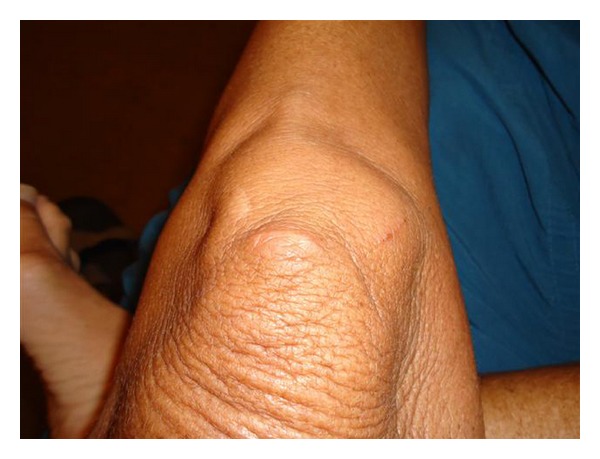


In July 2011, 11 years after the procedure, the patient presented edema and nodules with a petrified consistency in the areas where the substance had been injected (Figures 1 and 2). In addition, she presented infiltration of a prior scar on the left knee, making it hypertrophic (Figure 3). The patient was psychologically affected not allowing a skin biopsy.

In October 2011, the patient went to our dermatology clinic, where she was diagnosed with an allergic granulomatous reaction to the filler substance.

**Figure 4:**
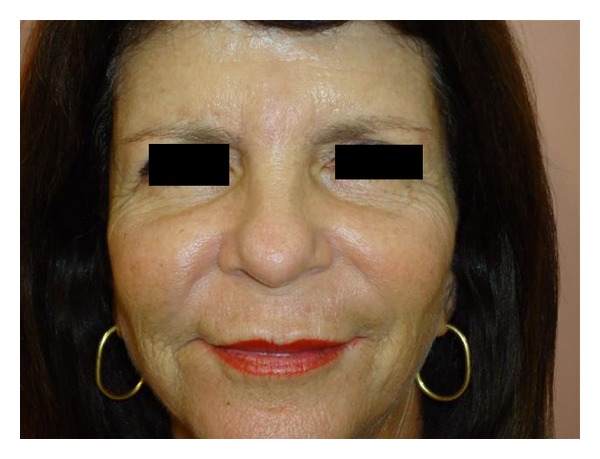


Treatment was initiated with allopurinol orally at a dose of 200 mg/day for 1 month. On the second month the dose was increased to 300 mg/day and maintained for 3 more months. There was significant healing of the lesions since the first month of treatment (Figure 4), returning to its physiological state according to the patient.

The patient returned for a followup, 3 months after the treatment with allopurinol had ended and there were no signs or symptoms of a possible relapse.

The patient was then referred to undergo head and neck surgery at the National Institute of Traumatology and Orthopedics, where she went through an MRI, which identified the well-limited sites of the application of PMMA (Figure 5) and is currently in preoperative evaluation for serial excision of the injected material.

## 3. Discussion

Produced since 1902, the substance PMMA was used initially for bone cement [4]. Nowadays, the main use of such a substance is to correct the nasolabial folds, as well as being indicated in patients with well-defined deeper facial wrinkle lines and little excess skin laxity [2]. The action mechanism involved in the desired effect of the filler is based on the degradation of the carrier collagen that occurs in about 1–3 months after the procedure. Thus, the new collagen produced has the ability to encapsulate the remaining particles of PMMA, since the nonbiodegradable properties and the substantial size of PMMA microsphere disabling the phagocytosis by macrophagesare noteworthy [2]. 

In a recent study, Salles et al. classified in five groups the possible complications caused by PMMA: among the 32 patients studied, 10 had palpable granulomas, showing that there is considerable risk for this complication to occur and it is not infrequent [1]. Despite the significant frequency of occurrence, the pathogenesis of granuloma formation has not been fully elucidated [5]. However, some theories have been proposed in order to partly explain such phenomenon: implant contaminated by impurities, application of excessive volume of the product in a single session, and nonhomogeneous surface of the PMMA particles [2]. It is important to emphasize that exogenous causes were also described and related to the formation of granuloma: infection [6], psychological shock, and surgical procedures [7]. 

Allopurinol is used as the standard treatment for hyperuricaemia and has been reported to be effective in sarcoidosis [3, 8, 9]. The exact action mechanism is still unknown [3, 8, 9]. However, some authors have speculated that allopurinol is an inhibitor of xanthine oxidase, a catalyst in the formation of superoxide [3, 8, 9]. Consequently, allopurinol and its metabolite, oxypurinol, act as free radical scavengers. Free radicals are supposed to play an important role in the pathogenesis of granulomatous diseases, and the reduction of their amount could be the key to the therapeutic benefit of allopurinol [3, 8, 9].

 Reisberger et al. treated a patient with foreign body granulomas caused by polymethylmethacrylate microspheres using allopurinol, showing great improvement of these granulomas [3]. 

Foreign body granulomas caused by fillers are usually observed near filler-injected areas, although they can also be found at distant sites [8]. Because of its impressive positive effect we decided to treat our patient with allopurinol. After 8 weeks of treatment, almost complete regression was seen in the local and distant granuloma, being successfully treated with allopurinol.

Due to a considerable number of incidents reported describing the appearance of granulomas in PMMA filled areas, and the positive outcome of the treatment with allopurinol in the case described previously, it could be safe to say that a study on this subject would be very instructive. A research could be conducted with as many volunteers possible to evaluate the success or failure, and how each individual and patient with different filled in areas react to the treatment. Since more and more people seek esthetical treatments with filling substances, like PMMA, this study could be very significant for medical purposes. 

## Figures and Tables

**Figure 5:**